# The Impact of Palliative Chemotherapy on the Survival of Patients With Metastatic Colorectal Cancer in Jordan

**DOI:** 10.7759/cureus.46187

**Published:** 2023-09-29

**Authors:** Mohammad S Alkader, Ahmed A Shahin, Mohammad S Alsoreeky, Hanna B Matarweh, Ilham A Abdullah

**Affiliations:** 1 Department of Medical Oncology, Jordanian Royal Medical Services, Amman, JOR; 2 Department of Internal Medicine, Arab Medical Center, Amman, JOR; 3 Department of Internal Medicine, Jordanian Royal Medical Services, Amman, JOR

**Keywords:** folfiri, folfox, metastatic, survival, palliative chemotherapy, jordan, colorectal cancer

## Abstract

Background

In Jordan, managing metastatic colorectal cancer (mCRC) is particularly complex, considering limited resources, access to advanced therapies, and unique patient demographics. Palliative chemotherapy, an approach aimed at relieving symptoms and improving the quality of life in patients with advanced cancer, including mCRC, has gained attention as a treatment strategy. While palliative chemotherapy may not aim for complete cancer eradication, it can extend survival, manage disease-related symptoms, and enhance the patient's overall well-being. However, deciding to pursue palliative chemotherapy for mCRC patients involves individual patient characteristics, performance status, disease aggressiveness, potential treatment-related adverse effects, and available healthcare resources. Given the need for region-specific insights into treatment outcomes, the proposed study seeks to investigate the impact of palliative chemotherapy on overall survival (OS), specifically within Jordan's healthcare landscape. Our study aims to showcase palliative chemotherapy's effectiveness on OS in first-line settings.

Materials and methods

This study is a retrospective analysis conducted at the Military Cancer Center (MCAC) in Jordan. It includes 73 patients diagnosed with mCRC between January 1, 2018, and January 1, 2020. Data were obtained from electronic medical records, and patients were monitored until June 10, 2023. Various patient characteristics were analyzed, including age, sex, primary tumor site, metastatic site, and treatment options for mCRC. The study evaluated the effectiveness of palliative chemotherapy in improving survival rates compared to BSC.

Result

We conducted a study with 73 participants, whose mean age was 60.37 ±13.5 years and a median of 63. Of these patients, 51 (69.9%) were male, and 22 (30.1%) were female. The primary site of the tumor was located on the left side in 32 patients (43.9%), on the right side in 26 patients (35.6%), and rectal cancer in 15 patients (20.5%). The most common site of the tumor was the sigmoid (17 patients, 23.3%). The liver was the most common site of metastasis (52 patients, 71.2%). Of the patients, 47 (64.4%) received palliative chemotherapy, while 26 (35.6%) were kept on best supportive care (BSC). Of those who received chemotherapy, FOLFIRI was administered to 32 patients (43.8%) and FOLFOX to 15 patients (20.5%). Based on the Kaplan-Meier curve, palliative chemotherapy patients had a significantly longer OS than those who only received BSC. Patients with palliative chemotherapy had a median OS of 12.4 months, while those who only had BSC survived for 5.3 months. The HR was 0.36 with a 95% confidence interval of 0.2-0.62, and the P-value was less than 0.001.

Conclusion

This study shows that palliative chemotherapy offers a notable advantage and a significant survival benefit compared to BSC.

## Introduction

Colorectal cancer (CRC) is the third most prevalent cancer among males and females worldwide, accounting for 10% of all new cancer cases. It is also the second most common reason for cancer-related deaths, accounting for 9.4%, according to GLOBOCAN 2020 [1]. CRC is a prevalent type of cancer in Jordan, ranking second in both males and females and accounting for 10.6% of all cancer cases, according to the Jordan Cancer Registry 2018 [2]. It is the second leading cause of cancer-related deaths, with 10.5% and 9% mortality rates for male and female patients, respectively [2].

At the time of diagnosis, metastatic disease is observed in approximately 20% of patients with CRC [3]. Previous studies from Jordan revealed low knowledge and awareness about CRC among the public [4,5]. These studies reported a lack of awareness about the alarming symptoms of the disease and the reluctance to seek medical help by the general public in Jordan. These factors, along with the potential of improper diagnosis by physicians, count for the delayed presentation and diagnosis of CRC nationally. Accordingly, there is an urgent need to promote public awareness about CRC through official healthcare registries and institutions.

The incidence rate of CRC in Jordan is relatively low in both sexes compared to developed countries [6]. The overall five-year survival rate for patients with CRC in Jordan was 58.2% [7]. The known risk factors that increase the risk of CRC are obesity, increased salt and red meat consumption, decreased physical activity, and smoking [8].

While the majority of CRCs are sporadic, 5-10% are caused by hereditary syndromes, and the two most common types are Lynch syndrome and familial adenosis polyposis, both of which are autosomal dominant [9].

It is recommended that all patients with mCRC be offered palliative chemotherapy, which can increase survival time by up to 20 months and is offered depending on the performance status of patients [10]. In treating patients with metastatic CRC (mCRC) when distant metastases and the primary tumor are operable, curative resection of the primary tumor is carried out, and the removal of distant metastases is also considered. For patients who cannot undergo surgery, palliative chemotherapy may be used, along with targeted therapy, such as bevacizumab and cetuximab, depending on the location of the primary tumor and molecular profile [11].

To date, there has been no investigation into the potential impact of chemotherapy on the survival rate of mCRC patients in Jordan. To fill this gap, we undertook a retrospective study at a single center in Jordan. We aimed to analyze the characteristics of a cohort of individuals with mCRC and assess the impact of chemotherapy on their OS in first-line treatment.

## Materials and methods

Study design

This retrospective study was conducted at the Jordanian Military Cancer Center (MCAC), focusing on patients diagnosed with mCRC, specifically adenocarcinoma. The study period spanned from January 2018 to January 2020, and patients were followed up until June 2023.

Patient selection

A total of 73 participants were included in the study. The inclusion criteria were as follows: (1) adult individuals aged 18 years or above; (2) chemotherapy-naïve patients with confirmed adenocarcinoma on histopathology and metastatic disease on imaging studies; (3) patients who received palliative chemotherapy as first-line treatment; (4) individuals demonstrating a favorable performance status as classified by the Eastern Cooperative Oncology Group (ECOG) scale within the range of 0-2; (5) normal liver and kidney function tests; and (6) blood parameters falling within the normal range.

Patients were excluded from the study if they met any of the following exclusion criteria: (1) insufficient or incomplete medical records pertaining to treatment or follow-up; (2) concurrent presence of another primary malignancy; (3) histopathological classifications other than adenocarcinoma (e.g., neuroendocrine or squamous carcinoma); (4) usage of biologic agents concurrently with first-line chemotherapy; (5) presence of severe acute or chronic diseases, such as cardiovascular disease or renal failure; (6) individuals categorized as having poor performance status (ECOG 3 and 4); and (7) receipt of fewer than four cycles of chemotherapy.

Data collection

Data were collected from the electronic medical records at our institution, MCAC. Data included patient-tumor characteristics such as age, sex, primary tumor location, chemotherapy regimen, and metastatic site. All patients included in the analysis were treated with mFOLFOX6 regimen or FOLFIRI therapy regimen as follows: mFOLFOX6 regimen (oxaliplatin 85 mg/m^2^ on the first day, folinic acid 400 mg/m^2^ on the first day, 5-fluorouracil (5-FU) 400 mg/m^2^ intravenous (iv) bolus on the first day, 5-FU 2400 mg/m^2^ 46 hours continuous infusion) was applied every two weeks. In contrast, the FOLFIRI protocol consisted of 180 mg/m^2^ iv on day one of irinotecan, 400 mg/m^2^ iv of folinic acid, 5-FU 400 mg/m^2^ (iv) bolus on day one, and 5-FU 2400 mg/m^2^ infusion over 46 hours. Treatment was repeated every two weeks.

Statement of ethics

The Pharmaceutical and Clinical Research and Studies Committee and Professional Ethics Committee in Amman, Jordan, approved the study design and protocol. The approval for this research was granted under the designation number 7/2023.

Statistical analysis

The data were analyzed using the Statistical Package for the Social Sciences (SPSS) version 22.0 (IBM Corp., Armonk, NY). Categorical variables are presented as frequencies and percentages, and continuous variables are presented as the mean± standard deviation, or the median and range when appropriate.

The chi-square test was applied to compare the differences between patient groups based on treatment as a dichotomous variable, as well as to compare between the primary site of the tumor and metastatic sites. The independent student t-test was used to compare the mean age between groups. Kaplan-Meier survival analysis and log-rank assessment were used to compare the OS for patients who received chemotherapy to those who received BSC. The hazard ratio (HR) was computed using the Cox regression model. A statistical significance was reached if the p-value was less than 0.05.

## Results

Description of the study population

A total of 73 patients with a confirmed diagnosis of mCRC were included in this study. The mean age at diagnosis was 60.4±13.5 years, and a median of 63, ranging from 20 to 89. Most patients were males (n=51, 69.9%). The primary site of the tumor was located on the left side in 32 patients (43.9%), on the right side in 26 patients (35.6%), and in the rectum in 15 patients (20.5%). The liver was the most common site for metastasis (52 patients, 71.2%), followed by lymph nodes (30 patients, 41.1%) and lungs (22 patients, 30.1%). Of the patients, 47 (64.4%) received palliative chemotherapy, while 26 (35.6%) received BSC. Of those who received chemotherapy, FOLFIRI was administered to 32 patients (43.8%) and FOLFOX to 15 patients (20.5%). A further description of the study population is shown in Table 1.

**Table 1:** Patient characteristics BSC: Best Supportive Care †Others include (bone, adrenal, ovaries, and bone marrow)

Patient characteristics		Frequency	Percentage
Sex	Male	51	69.9
	Female	22	30.1
Primary site of the tumor	Left	32	43.9
	Right	26	35.6
	Rectum	15	20.5
Specific site	Rectum	15	20.5
	Sigmoid	17	23.3
	Descending	8	11
	Splenic flexure	7	9.6
	Transverse	5	6.8
	Hepatic flexure	4	5.5
	Ascending	12	16.4
	Cecum	5	6.8
Treatment	BSC	26	35.6
	FOLFOX	15	20.5
	FOLFIRI	32	43.8
Metastatic site	Liver	52	71.2
	Llung	22	30.1
	Lymph node	30	41.1
	Peritoneum	22	30.1
	Others^†^	10	13.7

Association between the primary site of tumor and metastatic site

Metastasis to the lungs was found to occur more frequently in rectal cancer compared to left and right colon cancer (p=0.018). However, no significant differences were observed between the primary tumor site and other sites of metastasis (Table 2).

**Table 2:** Association between the primary site of the tumor and metastatic site * Significant n: number of patients, others (bone, adrenal, ovaries, and bone marrow) † Others include (bone, adrenal, ovaries, and bone marrow)

Metastatic site	Status	Left (n=32) (%)	Right (n=26) (%)	Rectum (n=15)(%)	Total (n= 73)	P-value
Liver	Absent	8 (38.1)	6 (28.6)	7 (33.3)	21	0.226
	Present	24 (46.1)	20 (38.5)	8 (15.4)	52	
Lung	Absent	25 (49)	20 (39.3)	6 (11.7)	51	0.018*
	Present	7 (31.8)	6 (27.3)	9 (40.9)	22	
Lymph node	Absent	19 (44.2)	15 (34.9)	9 (20.9)	43	0.987
	Present	13 (43.3)	11 (36.7)	6 (20)	30	
Peritoneum	Absent	21 (41.1)	19 (37.3)	11 (21.5)	51	0.784
	Present	11 (50)	7 (31.9)	4 (18.1)	22	
Others^†^	Absent	29 (46)	22 (35)	12 (19)	63	0.585
	Present	3 (60)	4 (40)	3 (30)	10	

Survival analysis

**Figure 1:**
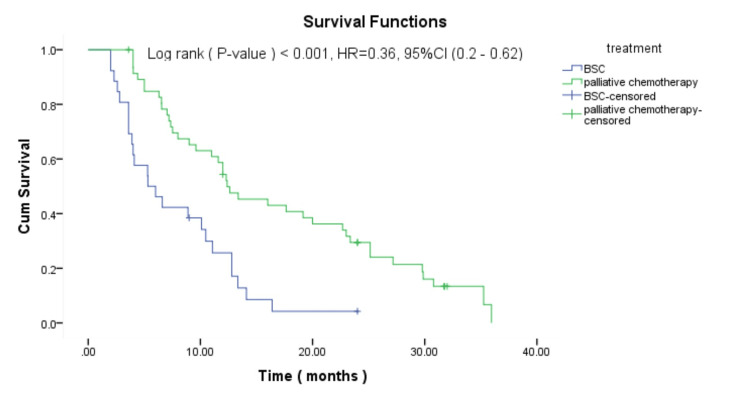
Kaplan-Meier curve for the overall survival according to treatment in mCRC patients CI: confident Interval; BSC: best supportive care; HR: hazard ratio; cum survival: cumulative survival Palliative chemotherapy: either FOLFOX or FOLFIRI

Survival analysis revealed that patients who received palliative chemotherapy in the first-line setting had a significantly longer median OS time of 13.7 months compared to a median survival of 5.3 months for those receiving BSC (p<0.001, HR: 0.36, 95%CI: 0.2-0.62). Figure 1 shows the survival curves of patients receiving palliative chemotherapy compared to those receiving BSC.

According to Table 3, there was no significant difference in baseline characteristics between patients receiving palliative chemotherapy and those receiving BSC, except for peritoneal metastasis, which was statistically different between the treatment groups (p=0.01). A greater proportion of patients with peritoneal metastasis received palliative chemotherapy (86.4%) compared to patients who received BSC (13.6%).

**Table 3:** Comparison of baseline characteristics for patients receiving palliative chemotherapy and patients receiving BSC Data presented as n (%) *Indicates statistical significance †Other sites of metastasis include the adrenals, ovaries, bone, and bone marrow BSC: best supportive care; SD, standard deviation

Characteristic		Chemotherapy (n=47)	BSC (n=26)	Total (n=73)	P-value
Age, mean±SD		58.4±12	63.9±15.4		0.09
Gender	Male	32 (62.7)	19 (37.3)	51	0.65
	Female	15 (68.1)	7 (31.9)	22	
Site of tumor	Left	31 (65.9)	16 (34.1)	47	0.70
	Right	16 (61.5)	10 (38.55)	26	
Liver metastasis	Absent	15 (71.4)	6 (28.6)	21	0.42
	Present	32 (61.5)	20 (38.5)	52	
Lung metastasis	Absent	35 (68.6)	16 (31.4)	51	0.25
	Present	12 (54.5)	10 (45.5)	22	
Lymph node metastasis	Absent	29 (67.4)	14 (32.6)	43	0.51
	Present	18 (60)	12 (40)	30	
Peritoneal metastasis	Absent	28 (54.9)	23 (45.1)	51	0.01*
	Present	19 (86.4)	3 (13.6)	22	
Others^†^	Absent	41 (65)	22 (35)	63	0.75
	Present	6 (60)	4 (40)	10	

**Figure 2:**
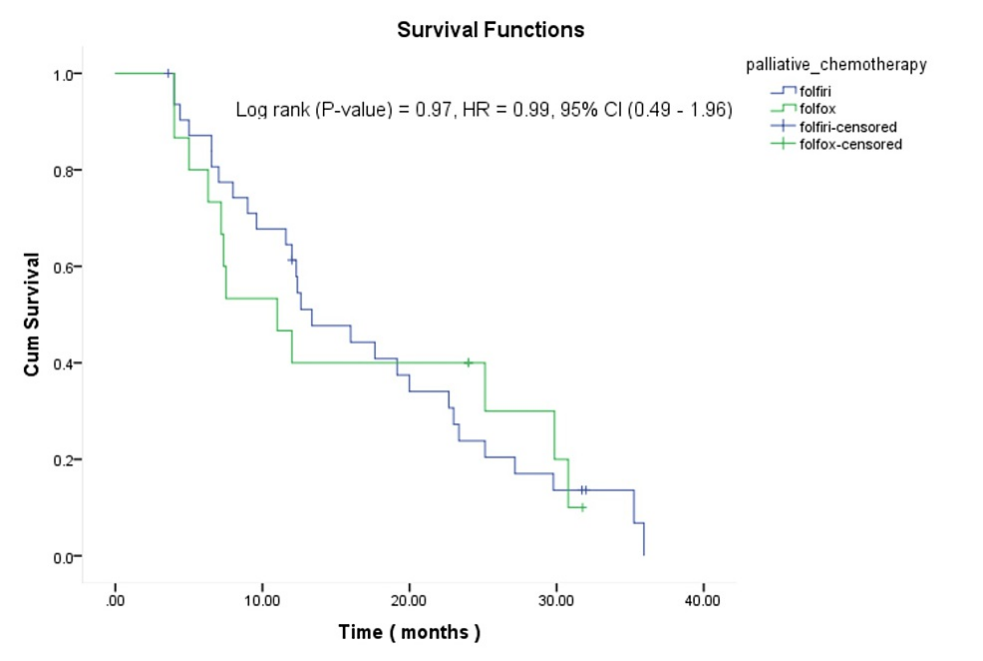
Kaplan-Meier curve for the overall survival comparing FOLFOX and FOLFIRI chemotherapy regimens in mCRC patients HR: hazard ratio; CI: confident Interval; cum survival: cumulative survival

Patients who were treated with either FOLFOX or FOLFIRI had comparable OS rates (median OS of 11 and 13.3 months, respectively). The survival rates for both regimens were not significantly different (p=0.97, HR: 0.99, 95% CI: 0.49-1.96). Figure 2 presents the survival curves for mCRC patients treated with FOLFOX and FOLFIRI regimens.

## Discussion

According to our research, the liver, lung, non-regional lymph nodes, and peritoneum were the sites most commonly affected by tumors. Additionally, rectal cancer was commonly metastasizing to the lung, whereas there was no significant association between liver metastases and the primary tumor site. A study conducted by the Korean Cancer Study Group CO12-04 revealed that the lung was the most frequently affected site for metastasis in rectal cancer. In contrast, liver metastasis was more commonly seen in left colon cancer [12].

It is essential to determine the goal of palliative chemotherapy in mCRC, which includes prolonging survival, improving cancer-related symptoms, and maintaining quality of life [13]. Our study showed that palliative chemotherapy, either FOLFOX or FOLFIRI, increased median OS compared to BSC (12.4 months vs. 5.3 months), respectively.

In a separate clinical trial, it was found that FOLFOX performed better than irinotecan plus bolus FU-LV (IFL) and irinotecan plus oxaliplatin (IROX) in terms of the median time to progression (8.7 months, 6.9 months, and 6.5 months, respectively), response rate (45%, 31%, and 35%, respectively), and median OS (19.5 months, 15 months, and 17.4 months, respectively) [14].

After analyzing SEER-medicare data to compare FOLFOX and FOLFIRI in first-line treatment for mCRC, it was found that there was no statistically significant difference in median OS between the two regimens. The FOLFOX arm had a median OS of 19.1 months, while the FOLFIRI arm had a median OS of 20.5 months (p=0.37) [15]. Our study yielded similar results, with no statistically significant difference in median OS between FOLFOX and FOLFIRI. The median OS for FOLFOX was 11 months, and 13.3 months for FOLFIRI (p=0.97).

Bevacizumab, also known as Avastin, is a monoclonal antibody used as the standard treatment for mCRC in both first- and second-line treatments. Its function is to target the vascular endothelial growth factor (VEGF). When added to the IFL regimen, it improves the median OS of patients, with 20.3 months, compared to IFL plus placebo, with a median OS of 15.6 months (p<0.001) [16].

Physicians who found it hard to access bevacizumab mentioned that the healthcare system or private insurance did not cover it and that patients had to pay high out-of-pocket costs. These barriers to accessing bevacizumab were especially prevalent among emerging market-based doctors [17]. In a randomized phase 3 clinical trial that compared FOLFIRI plus cetuximab and FOLFIRI plus bevacizumab, it was found that cetuximab improved survival rates in patients with left-sided primary tumors who had RAS wild-type. The study was conducted in a first-line setting, and the median OS in the cetuximab group was 31 months, while it was 26 months for the bevacizumab group (HR=0.76, 95% CI=0.62-0.94) [18].

Limitations

The study's retrospective nature and its confinement to a single-center setting constrain the generalizability of the findings. Data acquisition relies upon electronic medical records, which, while efficient, can harbor inconsistencies, gaps in information, and potential inaccuracies.

It is important to note that, while the study predominantly centers on OS as the primary outcome measure, other pertinent endpoints, such as quality of life, disease progression, and treatment response, were not explored in-depth. These additional dimensions could provide a more comprehensive understanding of the treatment's impact beyond mere survival.

## Conclusions

This study suggests that palliative chemotherapy provides a significant survival benefit compared to BSC. Although FOLFOX and FOLFIRI display similar OS rates, the analysis emphasizes the greater advantage of palliative chemotherapy over BSC in prolonging the lives of patients fighting advanced cancer. These findings offer valuable insights into clinical decision-making, highlight the significance of personalized treatment plans for patients with these challenging conditions, and contribute to the growing knowledge of mCRC treatment in the Middle Eastern region.
